# Mineralization of alpha-1-antitrypsin inclusion bodies in Mmalton alpha-1-antitrypsin deficiency

**DOI:** 10.1186/s13023-018-0821-7

**Published:** 2018-05-16

**Authors:** Francesco Callea, Isabella Giovannoni, Paola Francalanci, Renata Boldrini, Gavino Faa, Daniela Medicina, Valerio Nobili, Valeer J. Desmet, Kamal Ishak, Kuniaki Seyama, Emanuele Bellacchio

**Affiliations:** 1 Department of Pathology, Bambino Gesù Children’s Hospital, IRCCS, Piazza S. Onofrio 4, 00165 Rome, Italy; 2 Department of Cytomorphology, University of Cagliari, Cagliari, Italy; 3 Department of Pathology Spedali Civili, University of Brescia, Brescia, Italy; 4 Hepato-metabolic Unit, Bambino Gesù Children’s Hospital, IRCCS, Rome, Italy; 5 Department of Pathology, K.U.L. Leuven, Belgium; 6 Armed Forces Institute of Pathology, Washington, USA; 7 Division of Respiratory Medicine, Juntendo University Faculty of Medicine and Graduate School of Medicine, 2-1-1 Hongo, Bunkyo-Ku, Tokyo, 113-8421 Japan; 8 Genetic and Rare Diseases, Research Division, Bambino Gesù Children’s Hospital, IRCCS, Rome, Italy

**Keywords:** Alpha-1-antitrypsin deficiency, Mmalton, calcification

## Abstract

**Background:**

Alpha-1-antitrypsin (AAT) deficiency (AATD) of Z, Mmalton, Siiyama type is associated with liver storage of the mutant proteins and liver disease. The Z variant can be diagnosed on isoelectric focusing (IEF) while Mmalton and Siiyama may be missed or misdiagnosed with this technique. Therefore, molecular analysis is mandatory for their characterization. In particular, that holds true for the Mmalton variant as on IEF profile it resembles the wild M2 subtype.

**Methods:**

This is a retrospective analysis involving review of medical records and of liver biopsy specimens from a series of Mmalton, Z and Siiyama Alpha-1-antitrypsin deficiency patients. The review has been implemented by additional histological stains, electron microscopic observations and 3-D modeling studies of the sites of the mutations.

**Results:**

Z, Mmalton and Siiyama liver specimen contained characteristic intrahepatocytic PAS-D globules. The globules differed in the three variants as only Mmalton cases showed dark basophilic precipitates within the AAT inclusions. The precipitates were visualized in haematoxylin-eosin (H.E.) stained preparations and corresponded to calcium precipitates as demonstrated by von Kossa staining. On immunohistochemistry, ZAAT inclusions were stained by polyclonal as well as monoclonal noncommercial anti-AAT antibody (AZT11), whilst Mmalton and Siiyama inclusion bodies remained negative with the monoclonal anti-Z antibody. 3-D protein analysis allowed to predict more severe misfolding of the Mmalton molecule as compared to Z and Siiyama that could trigger anomalous interaction with endoplasmic reticulum chaperon proteins, namely calcium binding proteins.

**Conclusions:**

Mmalton AAT inclusion bodies contain calcium precipitates inside them that allow the differential diagnosis with Siiyama and ZAAT inclusions in routine histological sections. The study has confirmed the specificity of the monoclonal AZT11 for the Z mutant. Thus, the combination of these two features is crucial for the distinction between the three variants and for predicting the genotype, whose confirmation would definitely require molecular analysis. Our study provides new data on the pathomorphogenesis of Mmalton inclusion bodies whose mineralization could play a central role in disease pathogenesis of Mmalton that is distinct from the Z and Siiyama variants. Calcium is known to be a major effector of cell death either via the increased intracellular concentration or the alteration of homeostasis.

## Background

Alpha-1-antitrypsin (AAT) deficiency (AATD), an autosomal co-dominant genetic disorder, was discovered in 1963 on the base of the recurrent lack of alpha-1-globulin electrophoretic peak in members of the same family [1]. Subsequently, an analogous electrophoretic pattern was observed in children with cryptogenic liver cirrhosis whose hepatocytes contained peculiar PAS diastase (PAS-D) inclusions which reacted positively with a polyclonal anti AAT antibody [2]. Later on it was demonstrated that the protein retained within the hepatocytes was affected by a mutation, i.e. the replacement of glutamic acid by lysine at position 342 (Glu342Lys) in exon V of the *AAT* gene [3]. On Isoelectric Focusing (IEF), the mutant protein showed a very slow migration and for that reason, it has been designated with the last letter of the alphabet, Z. This letter indicates the phenotype of the main variant of the protease inhibitor (PiZ). Following the demonstration that the mutant protein was retained within the endoplasmic reticulum as a consequence of an abnormal conformation of the molecule, AATD has become the prototype of a new group of diseases, the Endoplasmic Reticulum Storage Disease (ERSD) [4], designated also as Conformational Diseases (CD) [5]. Presently about 100 allelic variants of the *AAT* gene have been detected, a few of them having lower than normal serum levels in the absence of liver pathology. Only three variants, Z, Mmalton and Siiyama are characterized by liver storage of the protein and are at risk of developing chronic liver disease and/or pulmonary emphysema. Other very rare variants have been shown polymerization capability in cell models [6, 7] but so far there are no observations in liver tissue specimens of those variants. The S mutation, which is the most frequent deficiency variant, is never resulting in liver storage [8, 9] most probably because of the instability of the molecule and its degradation before secretion [10]. The unique variant PiNull has low or no circulating AAT, no liver disease but pulmonary emphysema [11]. The discovery of AATD has led to the understanding of the pathogenesis of pulmonary emphysema as due to the proteolytic action of neutrophilic elastases on lung elastic tissue because of the deficiency of AAT, the major protease inhibitor (Pi). In contrast, the pathogenesis of liver damage is not completely known. The most plausible hypothesis refers to the more pronounced apoptosis of hepatocytes carrying higher amount of insoluble polymerized/aggregated AAT within the Endoplasmic Reticulum (ER), whilst the soluble forms are degraded by proteasome [12].

The observation that not all AATD individuals with hepatic storage develop liver disease, has favored the hypothesis that additional factors either environmental or genetic could be involved. Genes that negatively regulate autophagy, thus increasing the hepatocytic apoptosis, are potential candidates.

In the clinical setting, the characterization of AAT is requested in the presence of low serum levels of the protein. The first level investigation is IEF that helps in establishing the phenotype in most cases.

Previous study have shown that the serum concentration determination can be unreliable in heterozygous PiMZ phenotype as these individuals, under conditions of clinical stimulation, are capable of rising their serum levels up to the normal range due to the acute phase reactant nature of the M component. This phenomenon has been called “Recuitment-Secretory Block” phenomenon [13].

In addition, IEF has some limitations: Mmalton individuals show a mobility pattern analogous to the normal M2 subtype allele. With Mmalton, the situation becomes more complex when the mutation is associated with Z mutation, as the latter *per se* can explain the IEF pattern as well as the low serum levels. Finally, the Mmalton protein in homozygous condition can be visualized on IEF but it is quite indistinguishable from the compound Null/Mmalton condition [14].

The second level diagnostic investigation is molecular genetics, i.e. the sequencing of the entire gene of *AAT* that defines the genotype and identifies the pathogenetic mutations.

Z, Mmalton and Siiyama AAT accumulates in parenchymal liver cells and is visualized under the light microscope in the form of PAS-D inclusions that are positively stained by anti-AAT polyclonal antibodies.

Up to now, the three variants cannot be distinguished on the basis of the sole morphology. Moreover, one cannot rely upon epidemiological data that indicate a very high incidence (up to 7% in the general population) in Northern Europe [15]. Mmalton is much less rare but still is the most frequent variant in Sardinia [16], an Italian island with a high incidence of AATD deficiency. Siiyama is very rare and apparently confined to Far East [17, 18].

With regard to the immunological properties of the three variants, previous studies have proven that a noncommercial monoclonal anti-ZAAT antibody (AZT11) recognizes exclusively and selectively only the Z variant [15, 19, 20]. Recently Joly et al. [14] have reported a positive staining of AAT inclusions in a Mmalton/Mmalton patient using a not otherwise specified monoclonal antibody to AAT.

In this paper, we report on novel morphological findings that can enable to distinguish the most severe AATD variants, i.e. Z, Mmalton and Siiyama, and to unravel the pathogenesis of the liver cell damage.

## Methods

### Study Groups

- Group 1 (Mmalton patients)

Group 1 consisted of seven patients (Table 1), four adults and three children, undergoing a liver biopsy because of chronic elevation of liver enzymes and low plasma levels of AAT. The patients showed accumulation of AAT within hepatocytes and, on molecular analysis, displayed the Phe52deletion (Mmalton) either in heterozygous or in homozygous condition. In patient n.7 the liver biopsy had been performed after the genotyping, carried out first because of persistent very low serum AAT levels.

**Table 1:** Main clinical and laboratory findings in Mmalton cases

Case	Age	Sex	AAT level	Liver histology	Calcification	Genotype
1	38	F	20-75^a^	cirrhosis	+++	Homozygous
2	50	M	128^a^	cirrhosis	++	Heterozygous
3	62	M	109^a^	Cirrhosis and HCC	+++	Heterozygous
4	56	M	49^a^	cirrhosis	+++	Homozygous
5	4	M	75^b^	Mild CAH	++	Heterozygous
6	6	M	45^b^	Mild CAH	+++	Homozygous
7	2	M	20^b^	NSRH	–	Homozygous

^a^Radial immunodiffusion (n.v. 200–400 mg/dL); ^b^Nephelometry (n.v.90–200 mg/dL); *CAH* = Chronic Active Hepatitis; *NSRH* = Non Specific Reactive Hepatitis

The main clinical-pathological features of Group 1 patients are summarized in Table 1.

- Group 2 (Z patients)

Group 2 consisted of liver biopsies from seven patients (four adults and three children) diagnosed as Z AATD. Clinical-pathological features of Z patients are summarized in Table 2.

- Group 3 (Siiyama patient)

**Table 2:** Main clinical and laboratory findings in Z AAT patients

Case	Age	Sex	AAT level	Liver histology	Calcification	Genotype
1	22	M	28^a^	cirrhosis	–	Homozygous
2	44	M	82^a^	cirrhosis	–	Homozygous
3	45	M	75^a^	cirrhosis	–	Homozygous
4	56	F	102^a^	Cirrhosis and HCC	–	Compound heterozygous (S/Z)
5^c^	11	F	125^b^	PSC	–	Heterozygous
6^d^	17	M	196^b^	Mild CAH	–	Heterozygous
7^d^	13	M	222^b^	NSRH	–	Heterozygous

^a^Radial immunodiffusion (n.v. 200–400 mg/dL); ^b^Nephelometry (n.v.90–200 mg/dL); ^c^*PSC*: Primary sclerosing cholangitis; ^d^Liver biopsy obtained during a staging for Hodgkin’s lymphoma; *CAH* = Chronic Active Hepatitis; *NSRH* = Non Specific Reactive Hepatitis

Consisted of spared unstained histological sections from the Siiyama patient included in a previous study [19].

- Group 4 (Controls)

Liver biopsy specimens from two normal MM AAT individuals were used as controls.

The liver biopsies from the four Groups were obtained retrospectively.

### Morphological studies

#### Routine histology

Paraffin embedded liver tissue biopsy specimens were available from all Mmalton and Z AATD patients and from the Siiyama patient. The cases were selected on the base of the presence on routine histological examination, of eosinophilic PAS diastase resistant (PAS-D) inclusion bodies within hepatocytes.

Tissue sections were stained with hematoxylin-eosion (H.E.), PAS, PAS-D, Gomori’s reticulin, Masson’s trichrome, von Kossa for calcium and by immunohistochemistry with a polyclonal anti-AAT antibody. Additional serial sections were stained with a monoclonal antibody (AZT11) that recognizes specifically the Z variant as previously described [15, 19, 20].

#### Electron microscopy

A small fragment from needle or surgical specimens from all cases had been fixed in glutaraldheide and processed for transmission electron microscopy (TEM). Semithin sections were stained with toluidine blue. Ultrathin sections were mounted on copper grids, stained with lead citrate and uranyl acetate and examined with a Zeiss 109 TEM.

#### Molecular genetics analysis

DNA was isolated from total blood from all patients using extraction protocols from the QIAMP DNA mini kit (QIAGEN, Hilden, Germany), after the informed consent of patients or parents. Mutation screening was done using polymerase chain reaction (PCR), amplification and DNA sequencing of coding exons and all splices junction of *AAT* gene were carried out as previously described [20, 21].

#### Structural analysis

To represent the human AAT and to examine the amino acid environment around the sites affected by the Phe52deletion (Mmalton case) and Ser53Phe mutation (Siiyama), the Protein Data Bank (PDB) structure 1QLP was employed. The latent form of AAT was built by homology modelling using as the template the crystal structure of conserpin in the latent state (PDB entry 5CDZ). To display the Z form, the crystal structure of human AAT bearing the Glu342Lys amino acid change (PDB structure 5IO1) was employed.

The molecular volume of the crystal structure of the luminal domain of calnexin (PDB entry 1JHN) was used to infer the highest calcium concentration that can locally build up assuming tight aggregation of this protein and knowing that each molecule binds one Ca^2+^ ion.

## Results

### Morphological studies

#### Routine histology

**Fig. 1:**
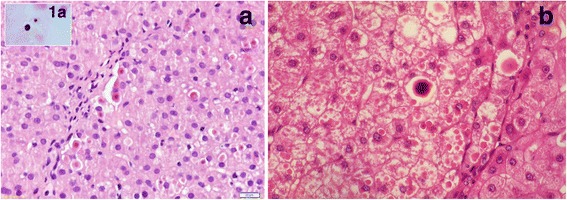
(**a**) Liver tissue section from case 6 Table 1 (Mmalton/Mmalton), with a preserved lobular architecture. Hepatocytes contain eosinophilic inclusions identified as AAT. A few of them mostly located in check-border hepatocytes are centered by dark basophilic material corresponding to calcium precipitates, H.E. 40X, positively stained by von Kossa staining, 100X (Fig. 1a, inset). (**b**) Liver tissue section from case 1 Table 1 (Mmalton/Mmalton), with fully established cirrhosis, H.E. 40X. The microphotograph shows hepatocytes plenty of cytoplasmic eosinophilic inclusions that were PAS-D positive and immunoreactive with a polyclonal anti-AAT antibody. The largest AAT globule is centered by a dark basophilic material, positively stained by von Kossa staining for calcium

On routine histology Mmalton, Z and Siiyama cases showed a variable degree of morphological alterations ranging from mild chronic hepatitis to fully developed cirrhosis, two of which complicated by hepatocellular carcinoma. These two cases were already reported for purposes other than the present study [21]. On light microscopy all cases presented round eosinophilic inclusions within hepatocytes. The inclusions were strongly PAS-D positive. In six out of seven Mmalton patients a number of AAT inclusions were centered by dark basophilic precipitates whose sized ranged from small (Fig.1a) to very large inclusions within AAT globules (Fig.1b). The dark basophilic material within AAT inclusions was not visible in PAS-D preparations but was positively stained by von Kossa staining for calcium (Fig.1a , inset). The number of calcified inclusions was scored semiquantitatively: + corresponded to 1–5% of periportal hepatocytes, ++ to 6–15%, +++ to 16–30%. The liver biopsy from case n.7 (Table 1) consisted of a very small and fragmented needle specimen. In this case, very few AAT inclusions devoid of basophilic precipitates could be found. Calcium precipitates were not seen in any ZAAT nor in the Siiyama case. On immunostaining, the Mmalton and Siiyama inclusions were positively stained by the polyclonal AAT antibody but remained negative with the monoclonal anti-ZAAT antibody, whilst the Z inclusions were positively stained by both the polyclonal and the monoclonal ZAAT antibody as previously described [9, 19, 20]. No AAT inclusion bodies or calcium precipitates were observed in MM normal control cases.

#### Electron microscopy

**Fig. 2:**
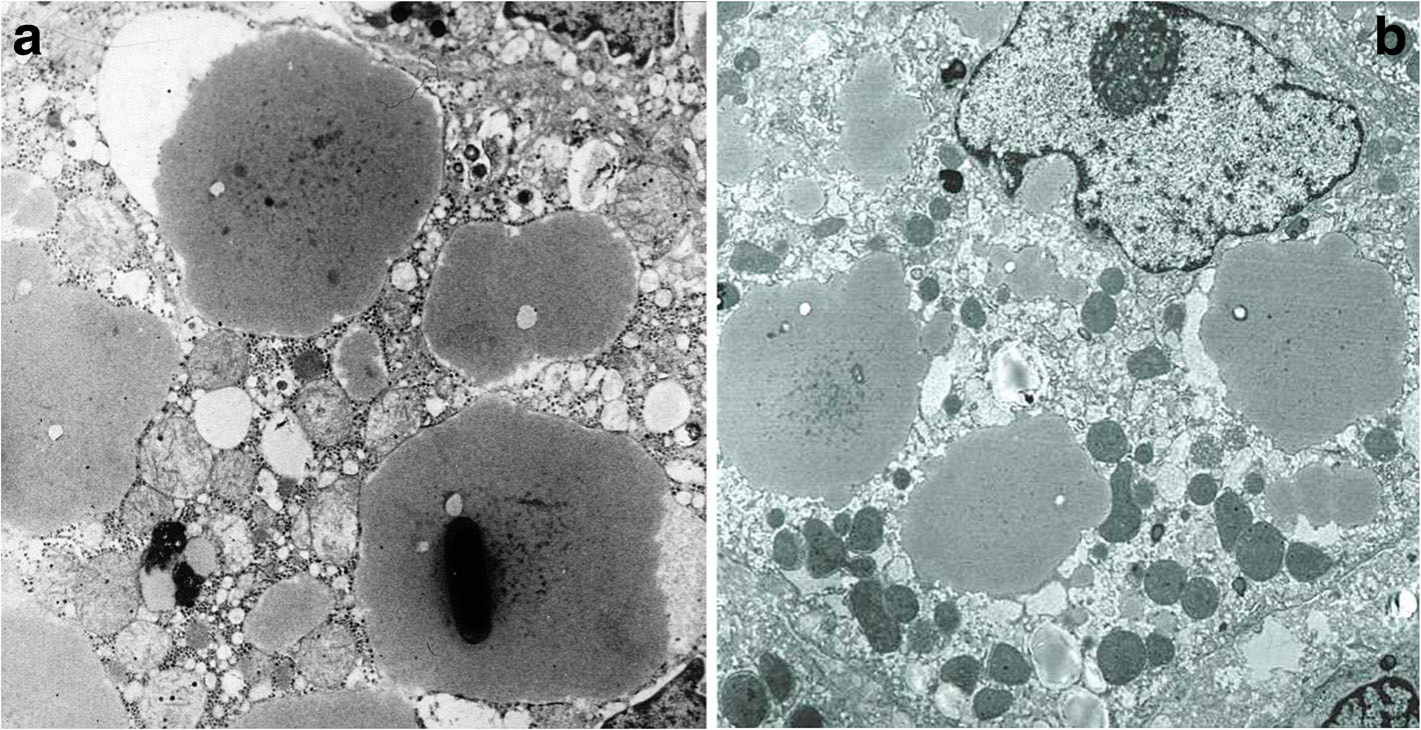
(**a**) Electromicrophotogaph from case 6 (Table 1) shows an hepatocyte with dilated cisternae of the ER containing fluffy semi-electron dense material corresponding to the classical appearance of AAT. The largest inclusions contain a large crystalline electrondense material. In addition to the crystalline structures, AAT inclusions contained fine or coarse electrondense granules quite similar to those of other AAT inclusions. This material was also displaying the peaks of calcium on EPMA (10.000 X). (**b**) Case 7 (a two years old boy with no visible calcium precipitates in H.E. stained preparations). The electronmicrophotograph shows dilated cisternae of ER. At least three AAT inclusions contain sparse electrondense granular precipitates similar to Fig. 2a. A few electrondense lysosomes with lipid inclusions are also seen (8000 X)

Under the EM, AAT material was localized in all cases within dilated cisternae of the ER. In six Mmalton cases, AAT inclusions displayed dark precipitates either in the form of granular non-crystalline or very electron dense crystalline material (Fig.2a). In the seventh Mmalton case (case n.7 Table 1) in which no calcium bodies were detected in H.E. stained preparations, fine electron dense granules quite similar to those of the other Mmalton cases were found in a few AAT inclusions (Fig.2b).

#### Structural analysis

**Fig. 3:**
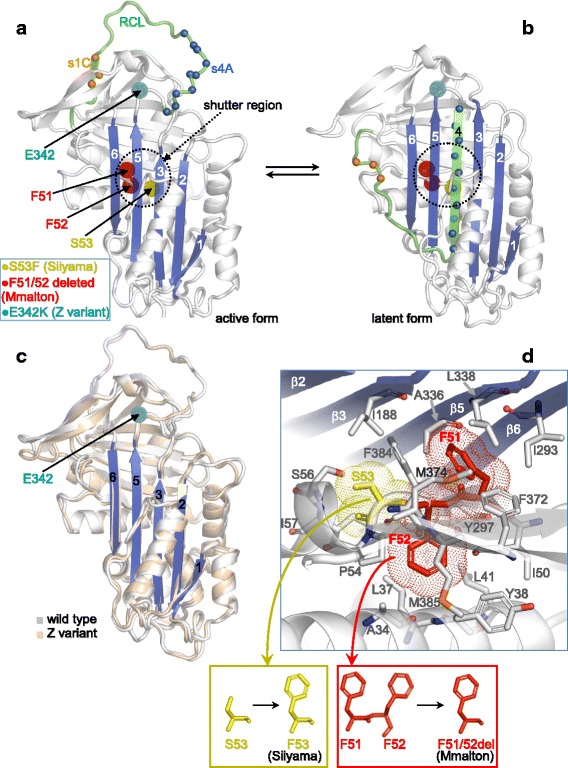
Sites of the Mmalton, Z and Siiyama mutations, and functional regions. (**a**) Active form of AAT structure (the β-strand region A is colored in blue). (**b**) Homology model of the latent form of AAT. (**c**) Superposition of the active AAT and Z variant structures. (**d**) Detailed view around the sites of Malton and Siiyama mutations highlighting surrounding core domain residues

AAT, as other serpin family members, presents an equilibrium between an active (Fig.3a) and latent (Fig.3b) form, with the latter derived from the former by relocation of the reactive center loop (RCL), which implies detachment of strand s1C from the β-sheet C region, and insertion of a portion of the RCL, structured as strand s4A, into the β-sheet A region between strands 3 and 5.

The Z variant is the unique hepatocytic storage variant that has been structurally characterized, and its Glu342Lys change has been proposed to induce lability in strand s5A, which produces AAT intermediates with only a partial insertion of this strand into the central beta-sheet A yet in equilibrium with the inhibitory native-like fold [22]. In fact, the Z form has been solved in a conformation that essentially overlaps with that of the active form of the wild type AAT (Fig.3c).

Although the structure of the Mmalton protein is still unknown, dramatic conformational changes can be envisaged for this AAT variant since the Phe52deletion occurs beneath the beta-sheet A region and results in the “coalescence” of Phe51-Phe52 pair of residues into a single phenylalanine with the alteration of several intramolecular interactions crucial for the domain core stability (Fig.3d). Concerning the Siiyama mutation, it also involves a residue beneath the β-sheet A (Fig.3d), but in this case the change consists of the missense Ser53Phe replacement, which destabilizes the affected region but not as much as the Phe52deletion in Mmalton.

## Discussion

Z, Mmalton and Siiyama deficiency variants of AAT are characterized by conformational abnormalities of amounts of the mutant molecules leading to insoluble polymerized/aggregated forms that accumulate in the ER of hepatocytes. Thus, the three variants fulfill the requirements for Endoplasmic Reticulum Storage Diseases (ERSD) [4] and Conformational Diseases [5].

AATD is worldwide distributed with a diverse incidence. The Z allele shows the highest incidence in the Northern Europe where it reaches about 7% of the general population [15]. The Siiyama is the most severe form of AATD in Far East [17, 18], Mmalton is the most common cause of AATD in the Italian island of Sardinia [16].

Individuals carrying the mutations present with reduced serum levels of AAT and accumulation of the mutant protein within hepatocytes.

The three forms are due respectively to the following mutations: Z to the missense Glu342Lys in exon V, Siiyama to the missense Ser53Phe in exon II and Mmalton to the Phe52deletion in exon II. The mutations are demonstrated by DNA sequencing of the entire *AAT* gene.

However, molecular genetics analysis is not available in all departments of Pathology, so that pathologists, in front of PAS-D hepatocytic inclusions, can only make the general diagnosis of AATD after proving that the stored material corresponds exclusively and selectively to AAT [4].

In this paper, we have provided a means to make feasible the differential diagnosis among the three variants in routine liver specimens. Indeed the three forms are indistinguishable on PAS-D staining and all react with polyclonal anti-AAT antibodies. However in contrast to the Z variant, Mmalton as well as Siiyama are not recognized by the monoclonal antibody (AZT11) that is specific for the Z variant [15, 19, 20]. Furthermore, only Mmalton presents with calcium precipitates visualized in H.E. stained preparations. This mineralization phenomenon does not occur with either Z or Siiyama AAT. Hence, it follows that the occurrence of calcium precipitates within AAT inclusions represents a diagnostic clue that *per se* would render unnecessary monoclonal antibody testing as well as molecular genetics analysis, especially if this technique is not available.

Molecular analysis remains the definite test also in Mmalton cases (like case n. 7 in our series) in which calcifications are not detected due to sampling error or insufficient tissue material.

To summarize, this study has unequivocally shown a striking difference between Mmalton, Z and Siiyama AATD in that Mmalton inclusions undergo a mineralization process and do not react with the monoclonal anti-ZAAT antibody. The two observations suggest the hypothesis that the two phenomena might have a relationship.

The monoclonal anti-ZAAT antibody appears to recognize a neoepitope lying close to position 342 and revealed upon formation of reactive loop β-sheet A polymers [19, 23, 24]. The negative staining of Mmalton inclusions with the Z antibody suggests that the structural perturbation leading to Mmalton polymers is different than that of the ZAAT polymerization. Indeed the Mmalton mutation has been shown to cause over insertion of the RCL and a likely C sheet mechanism of polymerization [23], whilst the Z mutation causes a partial insertion into β-sheet A, opening the lower part of β-sheet A, which then acts as an acceptor of the RCL of another ZAAT molecule [5, 23, 25].

In addition to definitely confirm in a large series of patients the impact of the Z mutation towards its immunological property, this study has led for the first time to the constant observation of calcium particles exclusively in samples from Mmalton patients, despite Z and Siiyama AAT presents similar fate of hepatocytic storage.

For the sake of completeness it has to be underlined that in our study we could examine a single Siiyama case and that one out of the seven reported Mmalton cases did not display calcifications on H.E. stained preparation.

However, to our knowledge, our Siiyama is the only one available case in the literature with liver histology. On the other side, the single Mmalton case from our study who did not show calcification on light microscopy, presented ultrastructural features quite analogous to the other six Mmalton cases. Once more, it is useful to emphasize that those features never occur in Z or Siiyama AATD variants. Of course, as calcifications of Mmalton globules represent a new observation, it would be important to check additional Mmalton livers as well as human liver tissue from other rare deficient variants capable of polymerization in vitro.

In order to explain the combination of the two phenomena observed in Mmalton, i.e. calcifications and lack of reactivity to the monoclonal ATZ11 antibody, we have inferred that Mmalton, among these three variants, is the most severely misfolded form of AAT and hypothesized that, in addition to hepatocytic storage, this mutant might also trigger damages by involving ER resident proteins. As a matter of facts, the ER functions as a storehouse of calcium ions, most of the resident proteins interact with AAT, and aberrant antitrypsin can activate endoplasmic reticulum-specific stress responses [26]. In this cellular defense mechanism, among the protein network evoked, calnexin, the major calcium transporter in the secretory pathway [27], which, in addition to binding calcium and regulating calcium homeostasis, has a molecular chaperoning function. Under stress, calnexin in *C. elegans* is present at higher levels and the overexpressed quote also binds calcium [28]. Furthermore, AAT is a well characterized cargo protein of calnexin [29], a physical association was seen between calnexin and a secretion-incompetent variant of human AAT [30], and calnexin was found to bind the alpha-1-antitrypsin null Hong Kong (NHK) releasing it, only once their interaction terminates, to the mammalian translocon-associated protein (TRAP) complex [31].

Concerning detrimental effects of AAT variants on the protein structure, it is worth to mention the work of Lomas et al. [23] who found that, compared to M or Z antitrypsin, the monomeric form of antitrypsin Mmalton had much slower rate in accepting an 11-mer antithrombin RCL peptide thus implying that this mutant harbors more important conformational changes in the A sheet region. The same authors also observed the unusual loss of the C-terminal peptide from the reactive loop-cleaved component of plasmatic antitrypsin, as this peptide normally remains tightly bound to the remainder of the domain.

On the other hand, biochemical indication that the defects of the Z form are much less severe and do not lead ineluctably to aggregations was provided by the evidences that the secretory impairment of Z antitrypsin can be corrected by chemical chaperons [32]. Furthermore, the insertion of the RCL peptide of one protein as strand s4A into the β-sheet A of another protein and so forth, proposed as mechanism of loop-sheet polymerization for aberrant antitrypsin forms, was successfully inhibited by introducing competitive exogenous RCL peptide fragments [33].

Finally, examination of the crystal structure of AAT in the active form shows that, among the Mmalton, Siiyama and Z mutation, the former (Phe52deletion) appears to be the most detrimental to structure integrity as it implies loss/alteration of several hydrophobic intramolecular interactions in the domain core beneath the β-sheet A region (Fig. 3).

This might seem at odds with the observation that the monomeric Mmalton apparently circulates in a normal functional form [23], but it is also true that antitrypsin inhibitory function relies on the flexible RCL peptide portion, which might remain available to its protease targets despite major rearrangements in the remainder of the AAT domain.

## Conclusion

Based on these evidences, it can be thought that ER stress and the associated calnexin upregulation triggered by Mmalton may lead to unusually strong interactions with this chaperone preventing it to escape after failed attempts to recover the AAT fold. In this hypothesis, Mmalton-induced aggregation would trigger the accumulation of calnexin thus the building up of calcium pools initially in protein-ligated form. Eventually, with the aging of the protein aggregates, free Ca^2+^ ions can be released fostering calcification. Abnormal aggregation of calnexin molecules can be a factor producing saturating levels of calcium. In fact, by assuming tight associations of calnexin (and taking into account the molecular volumes inferred from the crystal structure of the luminal domain of calnexin), the resulting concentration of calcium would be ca. 0.05 mol/L. This value exceeds the concentration necessary for the precipitation of calcium salts with physiologically available anions.

We believe that further investigations, including functional studies, are required to fully clarify the intriguing phenomenon observed in this study, especially with regard to the possibility of calnexin’s involvement.

Anyhow, the results appear to be relevant in that the mineralization of AAT inclusion bodies occurs only with the Mmalton protein thus allowing the unequivocal differential diagnosis between Mmalton, Z and Siiyama AATD and could play a central role in disease pathogenesis that is distinct from the common Z variant. Liver cell necrosis has longly been associated with intracellular calcium accumulation or its homeostasis alteration [34].

We feel that our observation can be implemented to expand the analysis of conformational diseases, in terms of pathological burden, and we hope that further studies will provide more meaningful arguments for the relevance of the novel data. Likewise, it is hoped that further studies, either retrospective or prospective, could be performed on as many as possible Mmalton livers especially from areas like the Mediterranean one, where the mutation is not so uncommon.
